# Short-Term Trend Forecast of Different Traffic Pollutants in Minnesota Based on Spot Velocity Conversion

**DOI:** 10.3390/ijerph15091925

**Published:** 2018-09-04

**Authors:** Xiaojian Hu, Dan Xu, Qian Wan

**Affiliations:** 1 Jiangsu Key Laboratory of Urban ITS, Southeast University, Nanjing 211189, China; 220162642@seu.edu.cn; 2 Jiangsu Province Collaborative Innovation Center of Modern Urban Traffic Technologies, Southeast University, Nanjing 211189, China; 3 School of Transportation, Southeast University, Nanjing 211189, China; wanqianhldcg@163.com; 4 Hualan Design & Consulting Group, Nanning 530011, China

**Keywords:** traffic emissions prediction, spot velocity, COPERT IV model, hybrid ARIMA model, emission control

## Abstract

Because traffic pollution is a global problem, the prediction of traffic emissions and the analysis of their influencing factors is the key to adopting management and control measures to reduce traffic emissions. Hence, the evaluation of the actual level of traffic emissions has gained more interest. The Computer Program to calculate Emissions from Road Transport model (COPERT) is being downloaded by 100 users per month and is being used in a large number of applications. This paper uses this model to calculate short-term vehicle emissions. The difference from the traditional research was that the input velocity parameter was not the empirical value, but through reasonable conversion of the spot velocity at one point, obtained by the roadside detector, which provided new ideas for predicting traffic emissions by the COPERT model. The hybrid Autoregressive Integrated Moving Average (ARIMA) Model was used to predict spot mean velocity, after converted it to the predicted interval velocity averaged for some period, input the conversion results and other parameters into the COPERT IV model to forecast short-term vehicle emissions. Six common emissions (CO, NO_X_, CO_2_, SO_2_, PM_10_, NMVOC) of four types of vehicles (PC, LDV, HDV, BUS) were discussed. As a result, PM_10_ emission estimates increased sharply during late peak hours (from 15:30 p.m.–18:00 p.m.), HDV contributed most of NO_X_ and SO_2_, accounting for 39% and 45% respectively. Based on this prediction method, the average traffic emissions on the freeway reached a minimum when interval mean velocity reduced to 40 km/h–60 km/h. This paper establishes a bridge between the emissions and velocity of traffic flow and provides new ideas for forecasting traffic emissions. It is further inferred that the implementation of dynamic velocity guidance and vehicle differential management has a controlling effect that improves on road traffic pollution emissions.

## 1. Introduction

The rapid increase in number of transport vehicles, especially for road transport, threatens sustainable development of our society in terms of energy consumption and traffic-induced pollutant emissions. Road transport is a significant source of air pollution [1]. People involved in motor vehicle emissions, such as policy makers, institutions, and the automotive and petroleum industries, are paying attention to environmental pollution problems in the world. Traffic emissions estimates are increasingly important for environmental policy assessments and infrastructure development [2]. Toxic gases and fumes emitted by vehicles can cause respiratory and cardiovascular diseases, nitrogen oxide (NO_X_) and volatile organic compounds (VOCs) can form ground-level ozone [3,4,5], a sign of photochemical smog pollution. Particulate matter (PM), also known as particle contamination, is a complex mixture of very small particles and droplets entering the air. Once inhaled, these particles can affect the heart and lungs and have a serious health impact [6,7,8]. Studies have found that vehicles contribute significantly to these emissions [9,10]. According to a review of the Convention on Long-range Transboundary Air Pollution (CLRTAP) data [11] by the European Environment Agency [12], road transport is the most significant source of NO_X_, CO, and Non-Methane Volatile Organic Components (NMVOCs) and the second most important source of PM_10_ and PM_2.5_ emissions. In 2006, only road transport accounted for 39.4% of total NO_X_ and 17.8% of total PM_2.5_ in 27 European Union countries (EU-27) [1]. Therefore, a variety of measures to control emissions have emerged. The federal or state has formulated a series of policies to regulate the pollution macroscopically [13]; the automobile industry is actively developing new energy-saving vehicles that meet environmental protection standards [14]. In addition to these measures, the introduction of the concept of traffic management and regulation is also an effective method to reduce emissions. Forecasting road traffic exhaust emissions in advance and discovering the influencing factors can help road traffic managers determine traffic management and control measures to effectively reduce vehicle emissions, especially toxic gas emissions.

In general, there are two typical models for estimating vehicle emissions: macro and micro models, which focus on different aspects of vehicle emissions calculations and analysis. At the micro level, the research focuses on the comprehensive model of second-by-second data based on different types of experimental vehicles to study the relationship between driving characteristics and emissions [15]. Representative models include MOter Vehicle Emission Simulator (MOVES), EL PASO Comprehensive Modal Emission Model (CMEM) and Passenger car and Heavy duty Emission Model (PHEM). Abou-Senna and Radwan [16] use VISSIM (from the German Verkehr In Städten-SIMulationsmodell) and MOVES to investigate the effect of major parameters on CO_2_ emissions. The results show that the consideration of the detailed microscopic analysis of vehicle acceleration and deceleration and its velocity has a significant impact on CO_2_ emissions. Liu et al. [17] used MOVES to estimate vehicle emission factor and concluded that different urban has different operating characteristics. MOBILE is an EPA emissions factor model for estimating pollution from on-road motor vehicles in states outside of California. MOBILE6.2 released in 2004 is the last version in that series. The MOBILE series has been superseded by the MOVES [18]. The model provides all inputs needed to discharge the air quality models. Although it is not a modal model, it is the most tested and validated model [19]. Shafie-Pourb and Tavakoli [20] used the International Vehicle Emissions (Ive) model to propose a bottom-up approach to assessing the total emissions of vehicles in the Iranian capital. Guevara et al. [21] stated that the total NO_X_ and VOC emissions simulated in the official Mexico City Metropolitan Area (MCMA) model are reduced by 37% and 26% when the MOtor Vehicle Emission Simulator for Mexico (MOVE-Mexico) was replaced by the Mobile Source Emission Factor Model for Mexico (MOBILE6.2-Mexico).

At the macro level, typical emission models, including the Computer Programme to calculate Emissions from Road Transport (COPERT), HBEFA v.3.1, and MOBILE, are commonly used for large-scale analysis to estimate total emissions and establish a city, state, or even national regional emissions inventories. Panis et al. [22] applied a comprehensive model to calculate instantaneous traffic emissions to actively manage vehicle travel velocities in urban networks. The results show that velocity management effectively reduces the average velocity of traffic; however, its impact on vehicle emissions is complex. Borge et al. [9] and Fontaras et al. [23] compared the road traffic emission in Madrid and European towns by the “average-velocity” COPERT4 and the “traffic situation” HBEFA models each other, which estimate road traffic emissions in Madrid and Europe. The former predicts total annual NO_X_ emissions is 21% higher than those of COPERT, and the latter has a good correlation in all cases. Lang et al. [24] estimated the emissions of road vehicles from 1999 to 2011 based on the COPERT model of vehicle emission factors with different emission standards, and analyze the spatial distribution of emissions and their relationship with Gross Domestic Product (GDP). Sun et al. [25] and Gong et al. [26] reported similar research. Quaassdorff et al. [27] simulated twelve different traffic conditions to estimate the corresponding NO_X_ and PM_10_ emissions in a hotspot with a high traffic density in Madrid (Spain). The results showed that local measures aimed at mitigating urban traffic congestion may have the potential to reduce emissions, and the estimated average emission factor estimated by the VISSIM-VERSIT [28] micro-modeling system is in good agreement with the emission coefficient of the average velocity model COPERT.

Some scholars estimate vehicle emissions through future weather forecasting models, such as “meteorological models”, which are specific to their respective research areas. Liu et al. [29] studied the changes in CO_2_ emissions under different weather conditions in different seasons of the year through the European emission factor model Assessment and Reliability of Transport Emission Models and Inventory Systems (ARTEMIS) [30].

In addition to the above model, there are other methods for detecting vehicle emissions. For example, to detect traffic emissions by using emission detection devices, at selected points in urban layouts away from hotspots. However, only a few countries, such as the U.S. and Europe have developed fairly accurate emissions projection tools. Simultaneously, limited monitoring stations may result in incomplete detection, the emissions measured by the monitoring station alone cannot provide an effective basis for controlling the vehicle emission.

Looking at the results of previous research, we can summarize the conclusions as follows: First of all, no matter which level is analyzed, because the input parameters of each model are different, there are more or less deviations from the results estimated by different models, making it difficult to verify and calibrate the estimated emissions, except the air quality monitoring area. Secondly, the average velocity is an important parameter for estimating vehicle emissions, but it is also related to other parameters such as traffic volume, regional vehicle operating characteristics, acceleration and deceleration changes, and road congestion levels. These parameters combined effects on vehicle emissions are complex. Third, the current research has no significant impact on the decision-making of exhaust emission measures, which is not conducive to traffic flow control and traffic management, to achieve vehicle guidance, and to effectively reduce emissions. Therefore, the current bottleneck is no longer to verify the reliability of the model, but to explore the control mechanism and influencing factors of road traffic emissions, and to find out the effective basis for reducing emissions. That is also the issue to be discussed in this paper.

From the point of traditional traffic control, with the goal of safety and high efficiency, when the speed is the fastest (in addition to Germany, generally below the speed limit), the overall traffic efficiency is the highest. However, the environment is the carrier on which human beings depend for survival. The pursuit of speed efficiency is at the expense of global environmental damage. Therefore, we should pursue the most suitable driving speed under the premise of aiming at minimum traffic emissions, this is from the perspective of exhaust control. The relationship between overall emissions and vehicle speed is parabolic. Minimum NO_X_ and CO_2_ when the speed is 20–80 km/h, other exhaust emissions show a one-way increase or decrease with speed [4,31]. Therefore, based on the superposition of all exhaust, when the speed is between 20–80 km/h, the total exhaust reaches the minimum. To guide and control the speed of vehicle under the premise that the total amount of road vehicles is minimized.

The purpose of this study is to use the COPERT IV model to predict the short-term vehicle emissions based on the spot velocity at one point obtained by a road detection device, and to provide management and control basis for controlling vehicle emissions. It introduces how to apply spot velocity to predict vehicle emissions, focusing on the interval average velocity conversion method and the other input parameters of the COPERT model. Taking the Minnesota highway system as an example, the results of vehicle velocity and emission forecasting are analyzed, the exhaust emissions of automobiles are analyzed, and a strategy of controlling vehicle exhaust emissions is proposed. This paper establishes a bridge between traffic emissions and vehicle velocity, and provides new ideas for predicting and controlling traffic emissions.

## 2. Methods

### 2.1. COPERT Model

COPERT is considered to be the standard emission inventory system for mobile sources. As a contribution to the Global Emissions Inventory Activity (GEIA), COPERT is also used to estimate emissions from road transport by many countries [32]. Ntziachristos et al. [33] used COPERT, HBEFA, and VERSIT+ to calculate the mean NO_X_ emission factor level. After comparing with the latest emission information collected by the laboratory experiment over real-world driving cycles [33], it was found that the Euro five-passenger car (PC) emission factor is a good reflection of the road environment of air pollution and collected the latest emissions information on the road using the Portable Emissions Measurement Systems (PEMS). Many other scholars have also applied the COPERT model to regional traffic emissions estimates in terms of one-year [24,25,26]. These studies have certain guiding significance for the assessment of regional traffic emissions.

**Figure 1:**
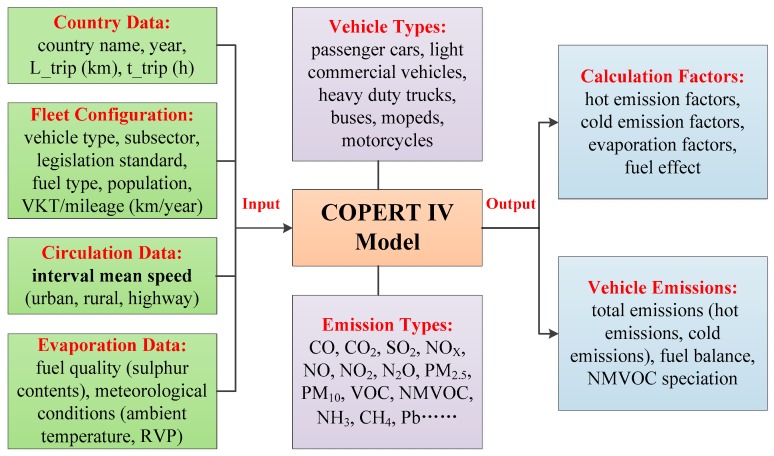
The specific input and output of COPERT IV model.

Most of the parameters required by the COPERT model are statistical. In addition, the interval mean velocity is a difficult to measure dynamic parameter. We will describe the method of obtaining the interval mean velocity in the next chapter. Other basic fixed input parameters for the COPERT model include vehicle type, emission standards, annual average vehicle mileage (VKT), vehicle population, fuel quality, and meteorological conditions, such as ambient temperature, Reid Vapor Pressure (RVP). Fixed data will be combined with a case studies and can generally be accessed by relevant authorities on the official website. Further conversion and matching are required to obtain vehicle types and emission standards, as different countries and regions have different requirements for vehicle models and emission standards. The specific inputs and outputs of the COPERT IV model are shown in Figure 1.

Vehicular emissions are calculated based on emission factors for each vehicle category and corresponding vehicle kilometers (VKT) using Equation (1):

(1) $$Q_{m,n}=\underset{i}{\sum }\underset{j}{\sum }\left(P_{m,i,j}\times VKT_{m,i}\times EF_{i,j,n}\right)$$

where *m* represents the area; *n* is the specific pollutants (i.e., CO, NMVOC, NO_X_, PM_10_, CO_2_, CH_4_, and N_2_O); *i* is the vehicle type, including passenger car (PC), light-duty vehicles (LDV), buses (BUS), heavy-duty trucks (HDT), and motorcycles (MC); *j* is the national vehicular emission standard (Euro I to Euro VI); $P_{m,i,j}$ is the number of vehicles in category *i* with emission standard *j* in states *m*; $VKT_{m,i}$ is the annual average vehicle kilometers travelled (km) for vehicles of category *i* in area *m*; and $EF_{i,j,n}$ is the emission factor (g/km) for pollutant n emitted from vehicles of category *i* with emission standard *j*. The emission factors are divided into hot emission factors and cold emission factors. The hot emission is, the engine and the aftertreatment devices have reached their normal operation temperature (i.e., they are hot). The other part refers to cold-start emissions.

### 2.2. Predict Interval Mean Velocity

As mentioned earlier, the dynamic input parameter of the COPERT IV model is the interval average velocity; therefore, the core of the predicted vehicle emissions is to predict interval average velocity. Currently, there are two expressions for determining traffic flow, spot mean velocity at one point (SMS, also known as time average velocity, measured with standard electromagnetic loops) and interval mean velocity (IMS, also known as space average velocity, in actual the IMS is used in the traffic flow model, corresponding to the average single link velocity between the vehicles provided by the GPS device). IMS is used in the actual traffic flow model, which is very important for accurate traffic modeling [34], but it’s difficult to obtain directly owing to technical reasons. Hallmark et al. [35] use the chase car method to collect the IMS along the arterial study links. This method works fairly well on arterial and lower-functional class roadways because there is a place to turn around and wait for the test car. The capacity of highway sections is limited, so this method is not applicable to traffic flow on highways. Sometimes (for example, in the case of a non-congested highway or in a situation where the road environment is less disturbed), it may be equivalent to SMS. However, this difference is significant when severely disturbed by the external environment, such as traffic accidents, bad weather or traffic flow is severely [36,37].

Therefore, considering that IMS is difficult to obtain directly as well as cannot be directly replaced by SMS, it is wise to obtain IMS through an indirect conversion. The focuses of this paper is based on the acquisition of spot speed at one point, transform it into interval speed, and then use it as an input parameter for emission prediction. This will be described in detail in the next chapter.

### 2.3. Predict Spot Mean Velocity

At present, there are many devices and equipment for collecting spot velocity on the road. Similarly, there exist many mature studies about predicting short-term traffic spot velocity. Vlahogianni et al. [38] presented a comprehensive literature review about short-term forecasting techniques up to 2003 and show that all the data intervals of highway flow or velocity vary between 20 s to 1 h, with 5-min being the most commonly used. Vlahogianni et al. [39] updated the literature review from 2004 to 2013. Chandra and Al-Deek [40] used auto-regressive integrated moving average (ARIMA) time series models to predict freeway traffic velocity and volumes and find that the past values of upstream as well as downstream stations influence the future values at a station and, therefore, can be used for prediction. Li and Rose [41] and Li and Chen [42] reported that the inclusion of rainfall (5 min data) on the short-term travel time predictions may reduce forecasting inaccuracies and improve model robustness. Zou et al. [43] use the hybrid short-term model to predict 5-min spot velocity and prove the approach can accommodate phase trends. Csikós et al. [44] adopted an artificial neural network to forecast velocity at the interval of 5-min, 15-min, and 30-min. The results show that the recognition rate of 5-min is higher than others, means that the prediction accuracy is higher with a five-minute interval. In summary, we use the hybrid ARIMA model considering upstream and downstream traffic flow to predict spot velocity, which will be suitable for predicting the spot mean velocity every five minutes. The mathematical representation of an ARIMA (*p*, *d*, *q*) model is as Equation (2):

(2) $$\varphi_{p}{\left(1-B\right)}^{d}X_{t}=\theta_{q}\left(B\right)\mu_{t}+\theta_{0}$$

where $X_{t}$ is the initial data set; $\mu_{t}$ is a white noise sequence that the mean is zero; variance is $\sigma^{2}$; *B* is the lag operators, $BX_{t}=X_{t-1}$; $\varphi_{p}$ is auto-regression operators, $\varphi_{p}\left(B\right)=\left(1-\varphi_{1}B-\varphi_{2}B^{2}-\cdots -\varphi_{q}B^{q}\right)$; q is order of moving average; $\theta_{0}$ is a parameter, $\theta_{0}=v\left(1-\varphi_{1}-\varphi_{2}-\cdots \varphi_{p}\right)$; and v is a mean value.

### 2.4. Velocity Conversion

What we predicted by the ARIMA model is spot velocity at one point, which does not directly correspond to average link velocity [45]. Therefore, in order to obtain the input parameters, it is a reasonable idea to convert the predicted spot average velocity to the interval average velocity as a predicted value. Because the input is the predicted speed, the strategy of predicting and then transforming can reduce the error. The spot velocity and conversion method is based on spot average velocity and interval average velocity, which is independent of actual or predicted values.

In the conversion process, in order to guide the velocity of the traffic flow and avoid excessive frequency induction, 30 min is a reasonable time to adjust the free flow velocity in advance. Therefore, in the second step, the predicted point velocity is averaged to obtain a predicted average spot velocity of 30 min. The specific steps are as follows. Firstly, the predicted velocity in 5 min is taken as the instantaneous velocity of each vehicle, then all instantaneous velocity in the next 30 min (10 consecutive 5 min) can be calculated. Next, the weighted average of the predicted interval average velocity must be obtained in the next thirty minutes. Last, the predicted interval mean velocity in the next 30 min can be converted. Rakha and Zhang [34] estimated the interval mean velocity through the variance of time mean velocity, which is a reasonable method for conversion. Results show that the error range of spatial mean velocity estimation is between 0–1%, with high conversion accuracy. According to the Rakha and Zhang [34] conversion method, see the Equations (3) and (4). In the formulas, $\bar{V_{t}}$ means the predicted spot mean velocity, $\bar{V_{s}}$ means the predicted interval mean velocity, and $\sigma_{t}^{2}$ means the variance. For $\bar{V_{s}}$ is numerically more biased towards lower speed vehicles, because the low speed vehicle takes up space for a longer period of time in a particular road length. It is derived that $\bar{V_{s}}$ is equal to $\bar{V_{t}}$ minus the ratio of the mean square error to the space. Therefore, the interval mean velocity can be obtained in the next thirty minutes:

(3) $$\bar{V_{s}}=\bar{V_{t}}-\frac{\sigma_{t}^{2}}{\bar{V_{t}}}$$

(4) $$\sigma_{t}^{2}=\frac{\sum_{i=1}^{n}{\left(V_{i}-\bar{V_{t}}\right)}^{2}}{n}$$

## 3. Case Study and Data

### 3.1. Static Input Parameters of the COPERT IV Model

#### 3.1.1. Vehicle Types and Emission Standards

First, motor vehicle registration data cannot be used directly in Equation (3). The European vehicle exhaust emission standard table is shown in Table 1. 

The difference between Euro V and Euro VI is mainly due to the different standards for nitrogen oxides. European vehicle emission standards are not completely consistent with those of the US. To address this issue, this paper referred to the US EPA standards for emissions and fuel consumption of different vehicle types [46] and then matched emissions standards between Europe and Minnesota (Table 2). Second, by referring to relevant information and making some assumptions, the North American vehicle type was matched with the vehicle classes in the COPERT IV model (Table 3).

#### 3.1.2. Vehicle Population, VKT, Fuel Quality, and Meteorological Conditions

The vehicle population and VKT data in 2015 on a highway in Minnesota were obtained from the U.S. Federal Highway Administration [47]. The total number of these five types of vehicles (PC, LDV, HDV, BUS, MC) on the highways of Minnesota is about 5.09 million and the proportions are 41%, 43%, 10%, 1%, 5%, respectively. The VKT is about 57,395 million kilometers. If the short-term traffic flow emission is to be predicted, the vehicle population is the detected or the predicted value. This paper adopted the numbers of detected traffic flows as the input value.

Minnesota road transport involves fuels such as gasoline, diesel, liquefied petroleum gas and gasoline alcohol. Since 2006, the EPA has gradually implemented stricter regulations to reduce the sulfur content of diesel fuel to 15 ppm. This fuel is known as ultra-low sulfur diesel (ULSD) [48]. The EPA specifies the volatility to reach the region (40 CFR 80.27(a)(2)(i)) for the Reid vapor pressure (RVP) standard set at 9.0 psi for certain designated nonattainment areas (40 CFR 80.27(a)(2)(ii)), the RVP standard is 7.8 psi, and Minnesota is the first. Thus, its RVP limit is 9.0 psi, which is about 62.069 KPa [49]. The cold emissions occur before the vehicle subsystems have reached their normal operation temperature. During this phase emissions are higher and strongly depend on ambient conditions [1]. Calculating cold emissions requires monthly maximum and minimum temperatures. The temperature in Minnesota during the study phase came from the National Weather Service.

### 3.2. Dynamic Input Parameters—Predicted Interval Mean Velocity

**Figure 2:**
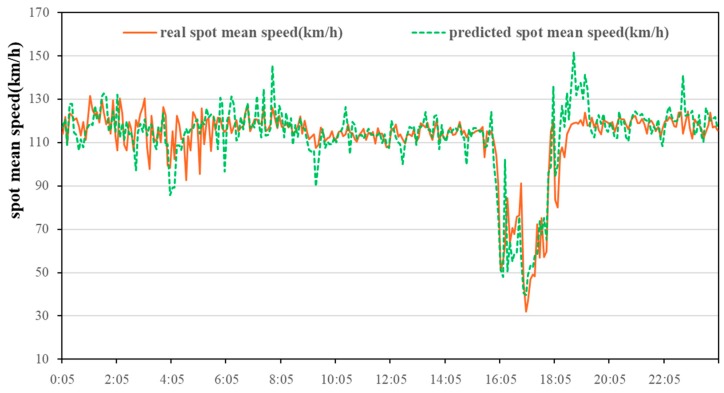
Spot mean velocity and predicted interval mean velocity on Minnesota highway.

**Figure 3:**
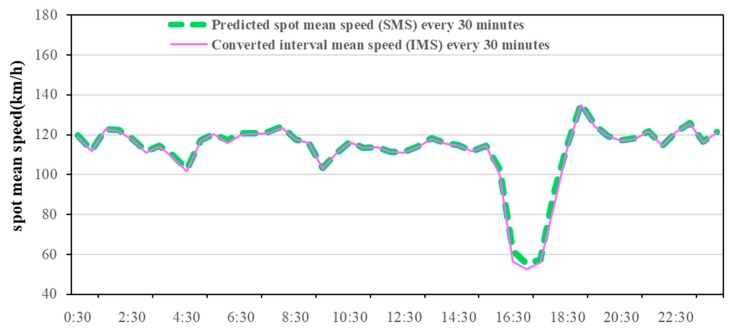
Predicted spot mean speed at (SMS) and converted interval mean speed (IMS) every 30 min.

The road traffic spot velocity was estimated on 14 July 2016, on the highway of I-394 in Twin Cities Metro area, Minnesota, located in North America. According to the method mentioned in Section 2.2, the 5-min spot velocity data of the I-394 five segments was obtained by the detecting device, and the validity was processed. Then the hybrid ARIMA model was used to predict the spot velocity of the five-segment intermediate segment within 5 min. The actual value was compared to the predicted value (Figure 2). The red solid line represents detected real spot mean velocity and the green dotted line represents the predicted spot mean velocity. Through velocity conversion, the final predicted daily interval average velocity every 30-min was obtained (Figure 3). The forecast velocity indicates that the morning peak of the Minnesota’s expressway is not obvious, but there is a clear late peak around 4:00–6:30 p.m., with the minimum interval average velocity is close to 40 km/h. These predicted interval average velocities will be used as input to the COPERT model to predict daily emission every 30 min.

## 4. Results and Discussion

The daily total emissions from the modelled traffic flow were predicted on a Minnesota freeway for 14 July 2016 (Figure 3). Because velocity prediction has little significance for the intelligent control of motorcycles (MC), there is no velocity and pollutant prediction for MC. This paper selected 12:00 p.m.–0:00 a.m., with the obvious fluctuation of velocity for analysis, and six common pollutants (CO, NO_X_, CO_2_, SO_2_, PM_10_, NMVOC) for analysis.

### 4.1. Emission of Different Pollutants

**Figure 4:**
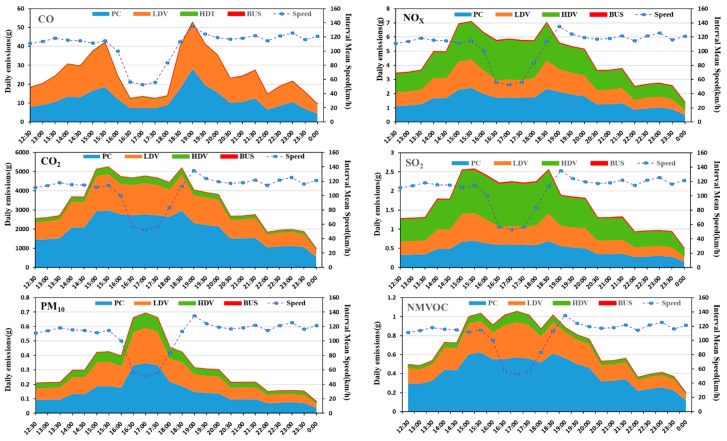
Daily vehicle emissions of different pollutants on Minnesota highway.

The predicted emissions of each pollutants depend on velocity (Figure 4). From the overall perspective, PC and LDV emissions accounted for 52% and 47% of the total emissions, with the large amount of CO emissions, accounting for 57% and 36% of total emissions respectively; emissions PM_10_ were the largest, accounting for total emissions 46% and 38% respectively; emissions NMVOC most, accounting for 59% and 33% of the total emissions, respectively. It was obvious that HDV contributed the most to NO_X_ and SO_2_, accounting for 39% and 45% respectively. PC and LDV accounted for NO_X_ 33% and 27% respectively, and SO_2_ 27% accounted for 27%.

**Figure 5:**
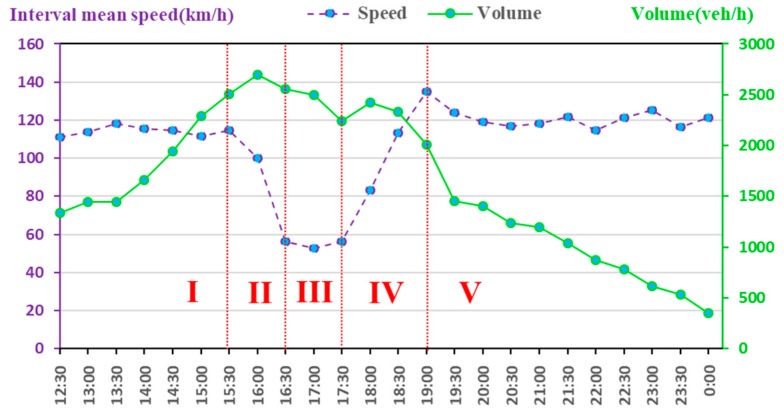
The dynamic variance of interval mean velocity and volume at different phase.

Based on the analysis of different pollutants, it is found that the exhaust emissions are not only relate to velocity but that they also have a close relationship with the traffic volume. We defined the peak hour in this situation as 15:30 p.m.–19:00 p.m. and, according to the velocity variance, five phases were divided to find out the related factors and regularity: the phase I was 12:00 p.m.–15:30 p.m., the phase II was 15:30 p.m.–16:30 p.m., the phase III was 16:30 p.m.–17:30 p.m., the phase IV was 17:30 p.m.–19:00 p.m., and the phase V was 19:00 p. m.–0:00 p. m. According to the prediction results, during the phase I, each emission substantially increased but the velocity is basically unchanged during this time. Therefore, the increase of exhaust emissions was caused by other factors (increased traffic volume) during the phase I, rather than velocity. Traffic volume was another important factor to study the traffic characteristics. According to the above analysis, the traffic volume data on the predicted phase was extracted for analysis together with the predicted interval mean velocity (Figure 5).

The following is a detailed analysis of each type of pollutant, as shown in Figure 4 and Figure 5. Figure 4 shown a large increase in all pollutants, indicating that traffic volume was an important factor in the increase in emissions. Figure 5 shows that during the phase I, the average velocity remained the same, but the traffic volume doubled. During the phase II, when the predicted interval mean velocity dropped to 54%, the traffic volume changed slightly, the CO dropped to 67%, and then remained at this level. NO_X_, CO_2_, and SO_2_ emissions had a similar trend to CO at the peak-time, but the decline is small, down about 15%, and then maintained at this level. The variance trends of PM_10_ and NMVOC were different from CO, both of them increased almost 75% during the phase II. During the phase III, the traffic volume, predicted average velocity, and emissions were almost invariable. During the phase IV, the predicted interval average velocity increased by 130% within one hour and then remained unchanged, traffic volume dropped by almost 49%, the CO increased sharply and substantially, with an increase of 250%. The NO_X_, CO_2_, and SO_2_ emissions had a similar trend to CO, but with a smaller ascent of about 22%. The change trends of PM_10_ and NMVOC were different from CO. Both dropped almost 35% during the phase IV and NMVOC had some fluctuations. During the phase V, all kinds of pollutants and traffic volume dropped; the predicted mean velocity almost remained unchanged.

**Figure 6:**
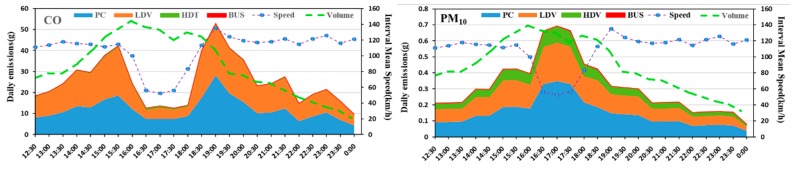
The relationship between CO and PM emissions with speed and traffic volume.

To this end, we further study the relationship between traffic volume with pollutants that vary significantly with speed. Here, the CO and PM_10_, which are more obvious and more harmful, are selected for analysis. As shown in Figure 6, some pollutants such as CO and PM_10_, relatively closely track the volume pattern and with minor effects from the change in speed during off-peak hours (during the phase I and phase V). However, at peak hours (during the phase II to IV), the relationship between CO and PM_10_ changes with traffic flow is significantly different. At this time, CO is positively correlated with speed, and PM_10_ is negatively correlated with speed, both of them are not significantly related to volume. That is because during this period, the road capacity is exceeded by the traffic volume, the emissions effects are thus consequences of the volume stress, and only addressing that volume of traffic will adjust the speed or volume. Therefore, in the early peak of pollutant release, some control measures can be taken to reduce the impact on the downstream by rationally guiding and controlling the upstream traffic flow. For example, for a frequently congested road section or area, in the pre-peak period, limit the number of vehicles or implement congestion charging to control the amount of traffic entering the congested area. 

**Figure 7:**
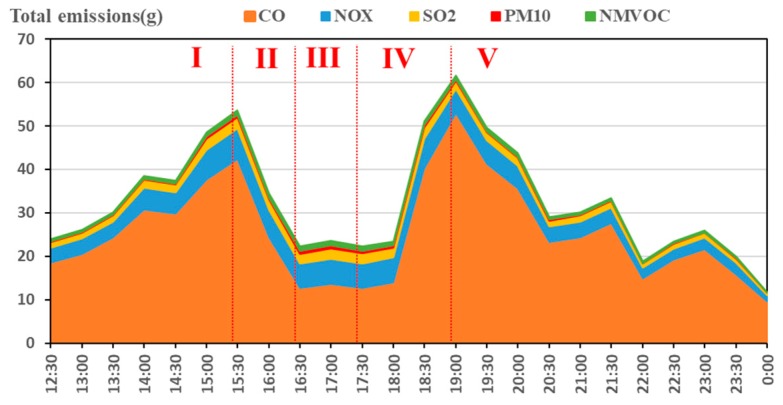
Dynamic variance of daily traffic flow total emissions on Minnesota freeway.

**Figure 8:**
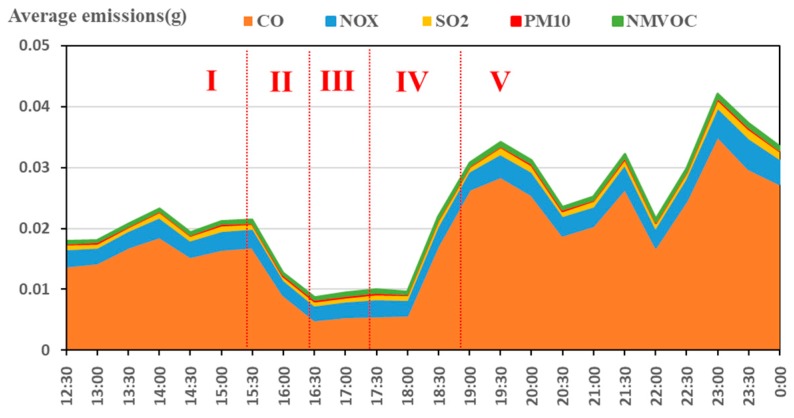
Dynamic variance of daily traffic flow **average emissions** on Minnesota freeway.

According to the above analysis, the pollutants increased in phase I and IV, and the actual traffic volume increased first and then decreased. The emission rules of different pollutants is different. When we control the pollutants, we may not only control one kind of pollutants, but pursue the overall optimality. Has the total emissions and the average emission inventories of the traffic flow increased? To find out the results, the dynamic changes in daily traffic volume total emissions and average emissions on the Minnesota expressway were shown in Figure 7 and Figure 8. The total emissions are based on the superposition of all exhaust, the average emissions are the average of the total emissions to all road traffic flows at different times. Since CO_2_ is less harmful to humans and emits much more than other pollutants, this part does not analyze CO_2_. As can be seen from Figure 7, the total emissions first increased during phase I and IV, decreased at the phase II and V, and remained at the lowest level in the phase III. However, there were some differences in the trends of the average emissions. The daily traffic flow average emissions remained unchanged during the phase I, and remained at the lowest level in the phase III.

From the above analysis one can see that when the interval average velocity of traffic flow is higher than 40 km/h, it was during the phase III (the interval average velocity was about 40–60 km/h) that the average emissions of traffic flow kept the lowest value, and during the phase IV (the interval mean velocity rose rapidly) that they increased substantially. According to the traffic flow theory, during peak-time, traffic volume on the road became gradually saturated, vehicles decelerated frequently, and the congestion queuing phenomenon appeared. After peak-time, the queuing vehicles’ acceleration began to dissipate, which led to the traffic flow average emissions increasing by a wide margin. The above conclusions are in line with Ritner et al.’s [50] research results, that acceleration emissions were in an order of magnitude larger than cruise emissions while deceleration emissions are smaller than cruise emissions. This does not mean that the lower the velocity, the less the exhaust emission. According to related research, the emissions of CO_2_, CO, and NO_X_ for individual drivers will rise sharply when the velocity is less than 40 km/h [1,51]. Carslaw et al. [52] used 20 instrumented vehicles with and without velocity control driven and found that the effect of mandatory ISA for petrol vehicles generally results in lower emissions of CO_2_, except for 20 mile/h velocity limits. That means it is useful to control driving velocity higher than 30 km/h from the perspective of environmental protection.

### 4.2. Emissions of Different Vehicles

**Figure 9:**
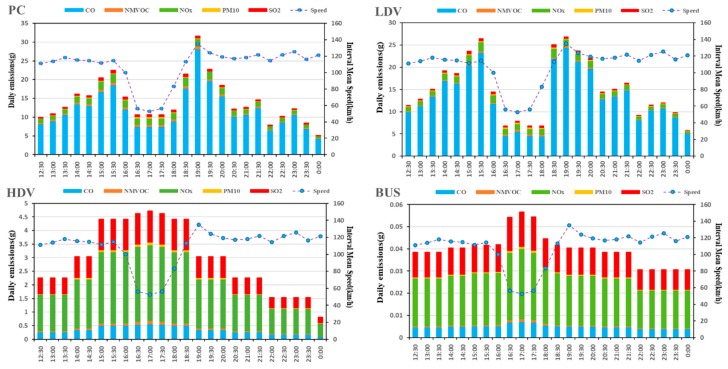
The dynamic variance of each vehicle emissions at each phase.

The dynamic variance of different vehicle emissions at different phases was shown in Figure 9. Similarly, CO_2_ is not analyzed here. On a whole, CO emissions from PC and LDV was high and, NO_X_ and SO_2_ emissions from HDV and BUS were high. During the phase I, PC and LDV increased to 38% and 45%, respectively; during the phase II, they decreased to 27% and 52%, respectively; and during the phase III, they increased to 45% and 95%, respectively again. HDV and BUS have different variations. During the phase I, HDV increased to 46%, was almost invariable during phase II to IV, and then decreased to 48% in the phase V. Emissions of BUS remained unchanged during the phase I, increased to 38% during the phase II and decreased to 15% during the phase V.

HDV and BUS exhaust emissions increased before or at peak-time (during the phase I to II) because they emit large amount of toxic gases such as SO_2_ and NO_2_. Sulfur dioxide and nitrogen oxides are fatal to children, especially when contaminants were not easily diffused in the air, which can exacerbate their toxicity and lead to a range of respiratory diseases. Therefore, such vehicles (HDV and BUS) are prohibited from driving on the road before peak hours and allowed to enter the ramp during off-peak hours. Implementing vehicle differentiation management can effectively reduce emissions of toxic gases.

## 5. Conclusions

This paper takes the daily average velocity and emissions (CO, NO_X_, CO_2_, SO_2_, PM_10_, NMVOC) of Minnesota highways as the research object, uses the COPERT IV model based on the field velocity to predict and analyzes different vehicle pollutants. With the rapid development of intelligent transportation, the proposed methods can be applied to highway velocity and emission prediction, to offer active velocity and traffic flow management on traffic-induced emission through an intelligent traffic system (ITS). This article draws the following four interesting conclusions.

Firstly, based on the spot velocity obtained by the roadside detectors, the short-term prediction of traffic exhaust emissions on the expressway section is realized. Vehicle exhaust is the main source of outdoor pollution. The calculation and prediction of vehicle emissions is an important issue for the transportation and environmental protection departments. The main dynamic input data of most emission models (such as COPERT model) is the interval mean velocity, and it is difficult to directly obtain and predict. Traditional studies have used empirical or estimated values, but the accuracy is low. At the same time, the device for getting the spot velocity on the road is more comprehensive, and the method of predicting spot velocity is relatively mature. Based on the availability of spot velocity, this paper predicts the spot velocity and converts it into the predicted interval average velocity, thus establishing a bridge between the spot velocity and vehicle emissions, providing theoretical basis for vehicle emission prediction.

Second, the variation of road traffic total emissions and average emissions in different phases of the day are different. However, when the interval average velocity is around 40–60 km/h, both reach or remain at the lowest level. The analysis results show that during peak hours (referred to as phase II to IV), the total traffic volume is basically unchanged, the interval average velocity drops first, remained low, and then rises. The total emissions and average emissions of traffic flow are similar to the velocity. These results help researchers to find a balance between traffic volume, velocity and emissions, take measures to control the impact of upstream flow on the downstream, keeping the velocity stable at a high level 30 min ahead (in this case, over 40 km/h), thereby inhibiting a significant increase in vehicle emission. This also indicates that we not only need to predict the velocity of vehicle, but also needs to predict the change of road traffic volume. Traffic volume is closely related to the emission and the application of the COPERT model.

Third, the relationship between vehicle velocity and different vehicular emissions provides a basis for differential guidance on different types of vehicles to reduce the emission of toxic gases. Through this study, HDV and BUS increased about 50% of toxic gases (SO_2_ and NO_2_) in phases I and II. Even a very small amount of these pollutants can cause great harm to human health and increase the risk of respiratory disease in children greatly. Therefore, not only does the traffic volume need to be controlled, but also measures must be taken to limit the vehicles (HDV, BUS) entering the congested roads during peak hours to avoid a sharp increase in emissions of such toxic gases. The toxic emission value can be used as an important influence factor affecting the road traffic impedance, and the original traffic impedance can be corrected to make a reasonable path planning.

Applying the emission prediction method in this study to the air quality model requires more information needs to be included in future works, such as establishing an air quality distribution model based on different roads and non-roads emission sources. At the same time, in order to reduce uncertainty, it is also necessary to establish and use more detailed traffic data based on the application value of emission data. Through prediction, the relationship between predicted interval mean velocity and vehicle emissions can be obtained in advance, and the velocity is related to the driving time or delay. Therefore, future research can consider the environmental costs caused by vehicle emissions and the travel costs caused by travel time (delay), thus establishing a dynamic velocity guidance model to minimize the social and economic costs.

## Figures and Tables

**Table 1:** European Stage X Motor Vehicle Pollutant Emission Standards Table.

European Standards	Gasoline Car Sulfur Content	Diesel Vehicle Sulfur Content	Implementation Time
Euro I	800 ppm/0.08%	2000 ppm/0.2%	1992
Euro II	500 ppm/0.05%	500 ppm/0.05%	1996
Euro III	150 ppm/0.015%	350 ppm/0.035%	2000
Euro IV	50 ppm/0.005%	50 ppm/0.005%	2005
Euro V	10 ppm/0.01%	10 ppm/0.01% (NO_X_ ≤ 180 ppm)	2008
Euro VI	10 ppm/0.01%	10 ppm/0.01% (NO_X_ ≤ 80 ppm)	2014

**Table 2:** Vehicle emission standards in Minnesota, North America, in 2015.

Vehicle Types	Euro I	Euro II	Euro III	Euro IV	Euro V	Euro VI
PC	✓	✓	✓	✓	✓	✓
LDV	✓	✓	✓	✓	✓	✓
HDT	✓	✓	✓	✓	✓	-
BUS	-	✓	✓	✓	✓	✓
MC	✓	✓	✓	-	-	-

**Table 3:** Vehicle categories used in North America and the corresponding categories in the COPERT IV model.

North America Vehicle Category	COPERT IV Vehicle Category
Light duty vehicles short WB 2/	Passenger car (PC)
Light duty vehicles long WB 2/	Light duty vehicles (LDV)
Pickup trucks	
Sport-utility vehicles	
Passenger vans	
Single-unit trucks 3/	Heavy-duty trucks (HDT)
Combination trucks	
Large pick-ups	
vans	
Truck tractors	
Recreational vehicles (RVs)	
Buses	Buses (BUS)
Motorcycles	Motorcycles (MC)
